# Efficacy and Safety of Adjunctive Corticosteroids Therapy for Severe Community-Acquired Pneumonia in Adults: An Updated Systematic Review and Meta-Analysis

**DOI:** 10.1371/journal.pone.0165942

**Published:** 2016-11-15

**Authors:** Jirui Bi, Jin Yang, Ying Wang, Cijiang Yao, Jing Mei, Ying Liu, Jiyu Cao, Youjin Lu

**Affiliations:** 1 Department of Respiratory Medicine, the Second Affiliated Hospital of Anhui Medical University, Hefei, China; 2 School of Public Health, Anhui Medical University, Hefei, China; 3 The Teaching Center for Preventive Medicine, School of Public Health, Anhui Medical University, Hefei, China; University of Leicester, UNITED KINGDOM

## Abstract

**Background:**

Adjunctive corticosteroids therapy is an attractive option for community-acquired pneumonia (CAP) treatment. However, the effectiveness of adjunctive corticosteroids on mortality of CAP remains inconsistent, especially in severe CAP. We performed a meta-analysis to evaluate the efficacy and safety of adjunctive corticosteroids in severe CAP patients.

**Methods:**

Three databases of PubMed, EMBASE and Cochrane Library were searched for related studies published in English up to December, 2015. Randomized controlled trials (RCTs) of corticosteroids in hospitalized adults with severe CAP were included. Meta-analysis was performed by a random-effect model with STATA 11.0 software. We estimated the summary risk ratios (RRs) or effect size (ES) with its corresponding 95% confidence interval (95%CI) to assess the outcomes.

**Results:**

We included 8 RCTs enrolling 528 severe CAP patients. Adjunctive corticosteroids significantly reduced all-cause mortality (RR = 0.46, 95%CI: 0.28 to 0.77, p = 0.003), risk of adult respiratory distress syndrome (ARDS) (RR = 0.23, 95%CI: 0.07 to 0.80, p = 0.02) and need for mechanical ventilation (RR = 0.50, 95%CI: 0.27 to 0.92, p = 0.026). Adjunctive corticosteroids did not increase frequency of hyperglycemia requiring treatment (RR = 1.03, 95%CI: 0.61 to 1.72, p = 0.91) or gastrointestinal hemorrhage (RR = 0.66, 95%CI: 0.19 to 2.31, p = 0.52). In subgroup analysis by duration of corticosteroids, we found that prolonged corticosteroids therapy significantly reduced all-cause mortality (RR = 0.41, 95%CI: 0.20 to 0.83, p = 0.01) and length of hospital stay (−4.76 days, 95% CI:-8.13 to -1.40, p = 0.006).

**Conclusions:**

Results from this meta-analysis suggested that adjunctive corticosteroids therapy was safe and beneficial for severe CAP. In addition, prolonged corticosteroids therapy was more effective. These results should be confirmed by adequately powered studies in the future.

## Introduction

Community-acquired pneumonia (CAP) is a common and serious infectious disease accompanied with high morbidity and mortality [1], about 20% of CAP adult patients acquire hospitalization, and has a mortality of 30–50% [2]. Furthermore, severe CAP occurs in approximately 10% of hospitalized patients with CAP, and has a higher mortality [3]. Despite recent advances in antimicrobial therapy and life-support measures, the mortality of severe CAP has not declined yet [4, 5]. Therefore, potential therapies of severe CAP should be explored.

Pneumonia is characterized by lung inflammation, with fluid filling the alveoli and preventing adequate oxygenation of the body [6]. During infectious pneumonia, an excessive release of circulating inflammatory cytokines, such as interleukin (IL)-10, IL-8 and IL-6 that acted as acute phase markers, would cause pulmonary dysfunction [7]. A recent study demonstrated that the raised levels of IL-6 and IL-10 were related with a high mortality in CAP, especially in severe CAP [8] which could potentially increase the incidences of sepsis, lung injury and acute respiratory distress syndrome (ARDS) [9]. Therefore, active and effective prevention of inflammatory deterioration is vital to severe CAP treatment.

Currently, corticosteroids are the most potent anti-inflammatory drugs. The therapeutic mechanism of corticosteroids might base on attenuating the action of many cytokines that participated in the inflammatory reaction associating severe CAP [10]. In addition, with the proposition of critical illness-related corticosteroid insufficiency (CIRCI), corticosteroids replacement therapy has been gradually accepted in critical illness, such as septic shock and ARDS [11]. Salluh et al. found that the most of patients with severe CAP could have relative adrenal insufficiency [12]. Another study also demonstrated that the reduction of baseline cortisol level could excerbate the severity and outcomes of severe CAP [13]. Up to date, many physicians have been using corticosteroids for severe CAP patients though the best usage regimens of corticosteroids were unclear [14]. Taken together, these facts indicated corticosteroids were of potential benefit in severe CAP.

In recent decades, adjunctive corticosteroids in severe CAP has been assessed in many randomized controlled trials (RCTs) [15–28]. However, the results of previous RCTs were inconsistent, and meta-analyses conducted to evaluate those RCTs had also failed to establish a complete agreement [29–37]. Recent meta-analyses conducted by Horita et al. [35] and Siemieniuk et al. [36] revealed that adjunctive corticosteroids statistically reduced the mortality of severe CAP. However, Wan et al. [37] proved that adjunctive corticosteroids was not associated with decreased mortality of patients with severe CAP. Therefore, we believed a meta-analysis, which depending on the best updating available evidence, was required. The aim of this study was to evaluate the efficacy and safety of adjunctive corticosteroids in the management of adult hospitalized patients with severe CAP.

## Materials and Methods

This study was conducted adhering to the Preferred Reporting Items for Systematic Reviews and Meta-Analyses (PRISMA) statement.

### Search strategy

We conducted a literature search in PubMed, EMBASE and Cochrane Library up to December, 2015. The search strategy included the following key words: “(severe community acquired pneumonia OR severe CAP) AND (corticosteroids OR corticotherapy OR steroids OR dexamethasone OR methylprednisolone OR prednisone OR cortisone OR hydrocortisone OR prednisolone)”. The search was limited to studies written in English and those utilizing RCT subjects. The detailed search strategy was available in S3 File. To ensure a comprehensive literature search, we also examined reference lists from included articles.

### Selection criteria

In this meta-analysis, we included studies that met the following eligibility criteria: (1) population: aged 18 years or older; (2) study design: RCTs (including quasi-RCTs), English language; (3) intervention: corticosteroids adjunctive therapy in severe CAP; (4) control intervention: placebo or standard treatment; (5) any types, any doses, and any durations of systemic corticosteroids were allowed; (6) primary outcome: all-cause mortality; (7) secondary outcomes: length of ICU stay, length of hospital stay, ARDS, need for mechanical ventilation and adverse effects. Articles were excluded based on the following criteria: (1) studies only had abstracts; (2) studies enrolled pediatric patients, nosocomial pneumonia patients or specified pathogens.

### Data Abstraction

Two reviewers extracted data independently and resolved disagreements by a consensus. Data was extracted from those included studies according to the pre-designed form: first author, year of publication, study design, location, number of patients, participant demographics, duration of corticosteroids treatment, doses of corticosteroids, evaluation criteria of severe CAP and outcomes. We contacted the authors of the included studies by E-mail if further study details were needed.

In previous meta-analyses, severe CAP was defined according to common criteria: CURB-65 (confusion, urea nitrogen, respiratory rate, blood pressure, and age 65 years or older) score of 2 or greater [38], Pneumonia Severity Index score (PSI) of IV or V [39], the American Thoracic Society (ATS) [40], and the British Thoracic Society criteria (BTS) [41]. The four proxies for “severe” CAP have advantages and disadvantages in clinical practice [42]. In addition, the two prediction tools SMART-COP [43] and predisposition, insult, response, and organ dysfunction (PIRO) [44] which are required further validation have similar sensitivity and specificity with ATS or outperform ATS [45]. In this study, we selected severe cases depended on the above tools, authors' classification of severe CAP when objective scoring was not available and limited to ICU cases.

### Data Synthesis and Statistical Analysis

If both per-protocol-analysis and intention-to-treat (ITT) analysis were conducted, ITT manner was chosen. Throughout the meta-analysis, we calculated risk ratios (RRs) and 95% confidence interval (CI) across all studies using a Mantel-Haenszel random-effects model for dichotomous outcomes and the effect size (ES) using an inverse-variance random effects model [46]. Heterogeneity was evaluated using the I^2^ test statistic and classified as no heterogeneity (I^2^ = 0), the least (≤25%), mild (25–50%), moderate (50–75%), and strong (≥75%) [47]. A sensitivity analysis was implemented by the sequential dropping of each study. The potential publication bias was assessed via funnel plots [48]. A p<0.05 was considered statistically significant. All statistical analyses were undertaken in STATA 11.0 software (Stata Corporation, College Station, TX).

## Results

### Trial flow

**Fig 1:**
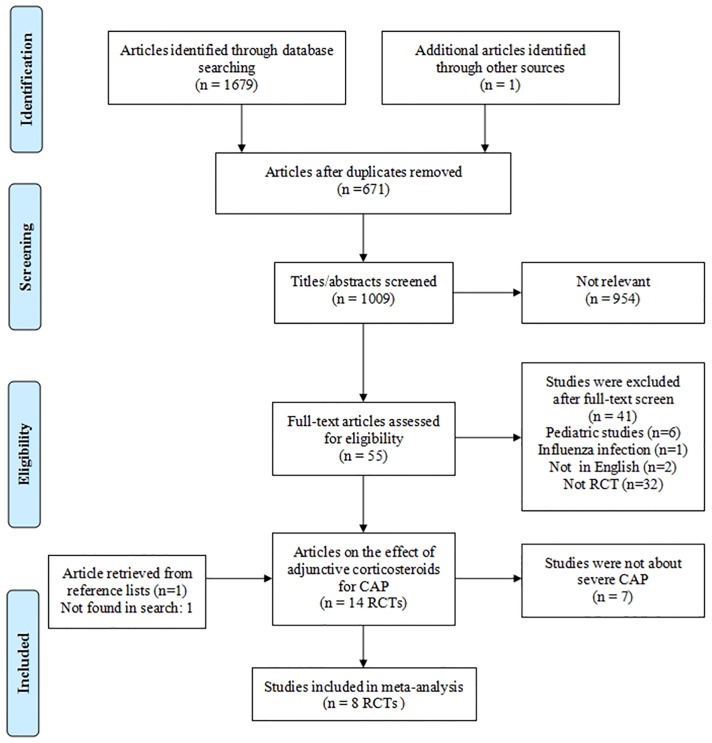
Flow diagram of the study selection process.

Our study design produced 1679 studies. Based on the inclusion and exclusion criteria, 1672 studies were excluded. Finally, 8 RCTs were included for meta-analysis [17–19, 21, 22, 24, 26, 28], and a PRISMA flow diagram of selection process for studies was shown in Fig 1. Among included RCTs, one study included mild to severe CAP patients, we selected the severe CAP cases which were defined by PSI of IV or V[21]. A study by El-Ghamrawy [19] which was not found in Search Databases, we obtained data from previous review [36].

### Study characteristics

Table 1 described the main characteristics of inculded studies, including 286 patients in the corticosteroids group and 242 patients in the control group. The different types of corticosteroids including hydrocortisone, methyl-prednisolone, dexamethasone and prednisolone. The durations of corticosteroids treatment ranged from 1 to 9 days. Various scoring systems, authors' classification of severe CAP and ICU cases were used to define severe CAP. Detailed extractive information from included studies were presented in Tables 1 and 2.

**Table 1:** Characteristics of included randomized controlled trials in the meta-analysis.

Study, year	Location	Study design	Sample sizes		Mean age (y)	Severe criterion	Corticosteroids used	Outcome
			n	N				
Marik [17], 1993	USA	DB RCT	14	16	34	ICU, BTS	Hydrocortisone, 10 mg/kg, 1d	Mortality
Confalonieri [18], 2005	Italy	DB RCT	23	23	64	ICU, ATS	Hydrocortisone, 240 mg/d, 7d	Hospital mortality
El-Ghamray [19], 2006	Saudi Arabia	DB RCT	17	17	61.8	ICU, ATS	Hydrocortisone 200 mg IV bolus followed by 10 mg/h for 7d	Hospital mortality
Snijders [21], 2010	Netherlands	DB RCT	48	45	63	PSI	Prednisolone, 40 mg/d, 7d	30-d mortality
Sabry [24], 2011	Egypt	DB RCT	40	40	62	ATS	Hydrocortisone, 300mg/d, 7d	ICU mortality
Fernandez-Serrano [22], 2011	Spain	DB RCT	23	22	63	PSI	Methylprednisolone, 620mg, 9d	Mortality
Nafae [26], 2013	Egypt	Open-label RCT	60	20	49	PSI	Hydrocortisone, 200mg (at day 1) and the 10mg/h IV for 7d	Mortality
Torres [28], 2015	Spain	DB RCT	61	59	65.3	ICU, ATS	Methylprednisolone, 0.5mg/Kg, 2/d, 5d	Hospital mortality

DB: double-blind; RCT: randomized controlled trial; ATS: American Thoracic Society; BTS: British Thoracic Society; PSI: Pneumonia Severity Index; ICU: intensive care unit; IV: intravenous. Severe community-acquired pneumonia is defined as PSI of IV or V, CURB-65 score ≥2; Meeting 1 of the ATS 1993 criteria; ATS 2001 rule where 1 major or 2 minor criteria are satisfied; ATS-IDSA 2007 rule where 1 major or 3 minor criteria are satisfied; BTS≥3. n: the number of patients in the corticosteroids group, N: the number of patients in the control group.

**Table 2:** Outcome data per study.

Study,Year	Sample size (n/N)	Mortality (n/N)	Treatment duration (n/N)	Length of hospital stay, d (n/N)	Length of ICU stay, d (n/N)	Developed ARDS (n/N)	Required mechanical ventilation (n/N)	Hyperglycemia requiring treatment (n/N)	Gastrointestinal bleeding (n/N)
Marik, 1993	14/16	1/3	≤5d	-	4.30±3.80/4.60±5.90	-	2/4	-	-
Confalonieri, 2005	23/23	0/8	>5d	22.25±10.75/29.25±17.25	14.25±7.25/21.00±10.25	0/4	-	-	1/1
El-Ghamray, 2006	17/17	3/6	>5d	-	-	0/3		-	1/1
Snijders, 2010	48/45	4/3	>5d	-	-	-		-	-
Sabry, 2011	40/40	2/6	>5d	-	-	2/6		-	-
Fernandez-Serrano, 2011	23/22	1/1	>5d	10.00±2.96/12±6.67	6.50±2.69/10.50±13.52	-	1/5	1/0	1/1
Nafae, 2013	60/20	4/6	>5d	9.27±2.40/16.50±2.24	3.10±4.90/6.30±8.20	-	8/5	19/8	1/1
Torres, 2015	61/59	6/9	≤5d	11.00±4.81/10.50±5.19	5.00±3.70/6.00±2.96	-	5/9	11/7	0/1

ICU: intensive care unit; ARDS: acute respiratory distress syndrome; n: the number of patients in the corticosteroids group, N: the number of patients in the control group.

### Quality Assessment

Two authors (Jirui Bi and Jin Yang) carried out data extraction and assessed the risk of bias independently, any conflicts were resolved with group consensus. The Cochrane Collaboration’s Risk of Bias Tool [49] was used to assess the quality of all included RCTs (Table 3).

**Table 3:** Summarizes the risk of bias.

Author	Randomization method	Allocation concealment	Blinding of participants and personnel	Blinding of outcome assessment	Incomplete outcome data	Selective reporting	Other bias	Overall risk
Marik	Low	Low	Low	Low	Low	Low	Unclear	Low
Confalonieri	Low	Low	Low	Low	Low	Low	Unclear	Low
El-Ghamray	Low	Unclear	Unclear	Low	Low	Low	Unclear	Low
Snijders	Low	Low	Low	Low	Low	Low	Low	Low
Sabry	Low	Low	Low	Low	Low	Low	Unclear	Low
Fernandez-Serrano	Low	Unclear	Low	Low	Unclear	Low	Low	Low
Nafae	Unclear	Low	Unclear	Unclear	Unclear	Low	Unclear	High
Torres	Low	Unclear	Low	Low	Low	Low	Low	Low

### Primary Outcome

The main results for binary outcomes (Mantel-Haenszel method, random effect model) were presented in Table 4.

**Table 4:** Main results for binary outcomes (Mantel-Haenszel method, random effect model).

Outcome	Studies, n	Pooled RR (or ES) (95% CI)	p-value	I^2^, %	p_heterogenelty_
All-cause mortality	8	0.46 (0.28 to 0.77)	0.003	0.0	0.481
Prescription duration					
≤5d	2	0.59 (0.24 to 1.43)	0.241	0.0	0.661
>5d	6	0.41 (0.21 to 0.82)	0.012	15.1	0.317
Length of hospital stay	3	-4.76 (-8.13 to -1.40)	0.006	43.4	0.170
Length of ICU stay	5	-1.84 (-4.23 to 0.56)	0.130	38.6	0.182
Developed ARDS	3	0.23 (0.07 to 0.80)	0.020	0.0	0.743
Required mechanical ventilation	4	0.50 (0.27 to 0.92)	0.026	0.0	0.841
Adverse effects					
hyperglycemia	3	1.03 (0.61 to 1.72)	0.91	0.0	0.385
gastrointestinal hemorrhage	5	0.66 (0.19 to 2.31)	0.52	0.0	0.954

RR: risk ratio; ES: effect size; ICU: intensive care unit; ARDS: acute respiratory distress syndrome.

### All-Cause Mortality

**Fig 2:**
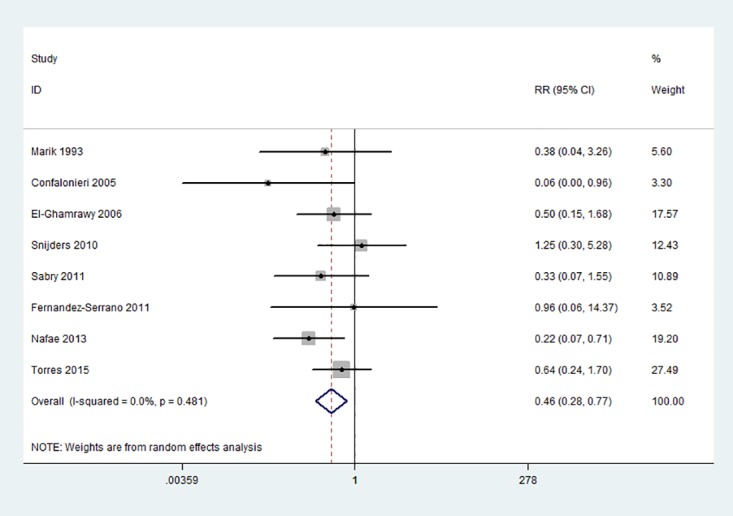
Forest plots of the association between all-cause mortality of severe CAP and corticosteroids.

The pooled results of all-cause mortality was significantly reduced by the use of corticosteroids (8 trials, RR = 0.46, 95%CI: 0.28 to 0.77, p = 0.003, I^2^ = 0%) (Fig 2).

**Fig 3:**
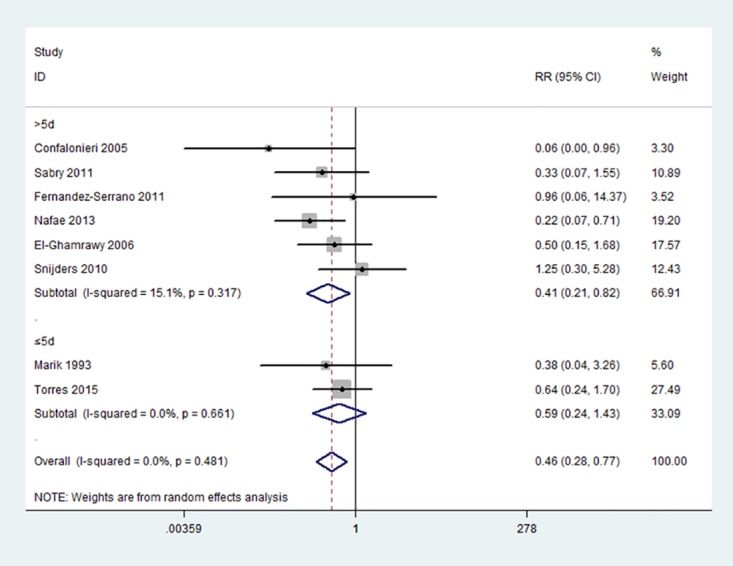
Subgroup analysis according to the duration of corticosteroids treatment in severe CAP.

Subgroup analysis was performed by duration of corticosteroids treatment. Corticosteroids therapy reduced all-cause mortality in severe CAP, but the best corticosteroids duration for severe CAP was not fully clear. Our analysis suggested that prolonged use of corticosteroids (>5 days) was more effective for severe CAP (RR = 0.41, 95%CI: 0.21 to 0.82, p = 0.01; I^2^ = 15.1%) (Fig 3). There was no statistically significant difference in short course of corticosteroids (≤5 days) (2 trials; RR = 0.59, 95%CI: 0.24 to 1.43, p = 0.241; I^2^ = 0%).

### Secondary outcomes

#### Length of hospital stay

**Fig 4:**
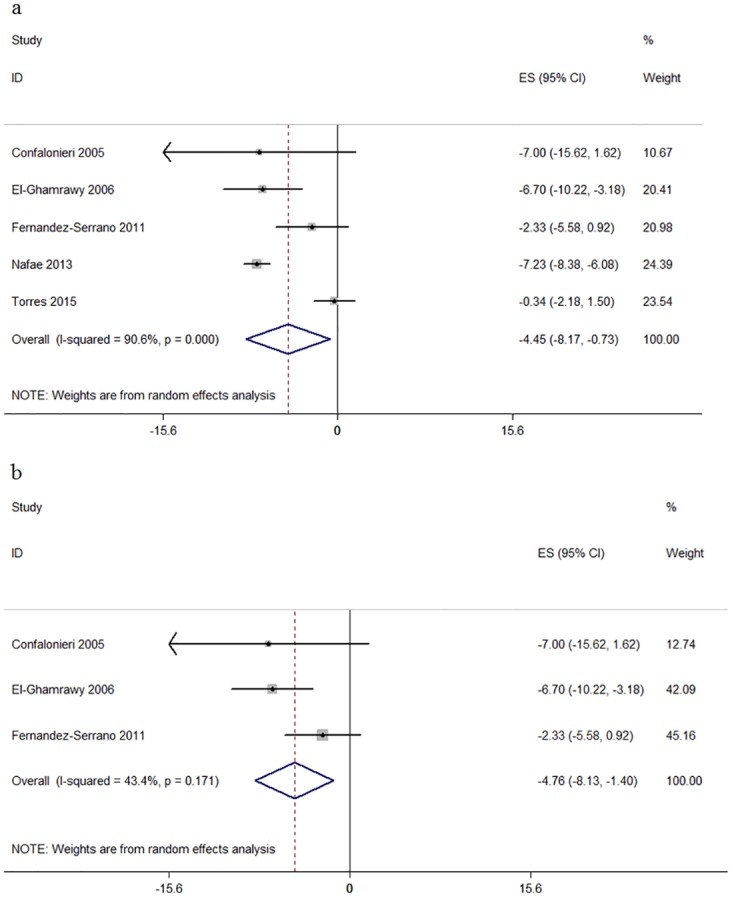
Forest plots of the association between length of hospital stay and corticosteroids.

5 RCTs reported the difference of length of hospital stay in days. Based on these data, we found strong heterogeneity (I^2^ = 90.6%) in the primary analysis of hospital stay days (Fig 4a). In subgroup analysis by the treatment duration of corticosteroids, the result was −5.79 days (95% CI:-8.17 to -2.31, I^2^ = 61.4%), there was also moderate heterogeneity. We performed a sensitivity analysis excluding Nafae, and the final result was −4.76 days (95% CI:-8.13 to -1.40, p = 0.006; I^2^ = 43.4%, p for heterogeneity = 0.17) (Fig 4b).

#### Length of ICU stay

**Fig 5:**
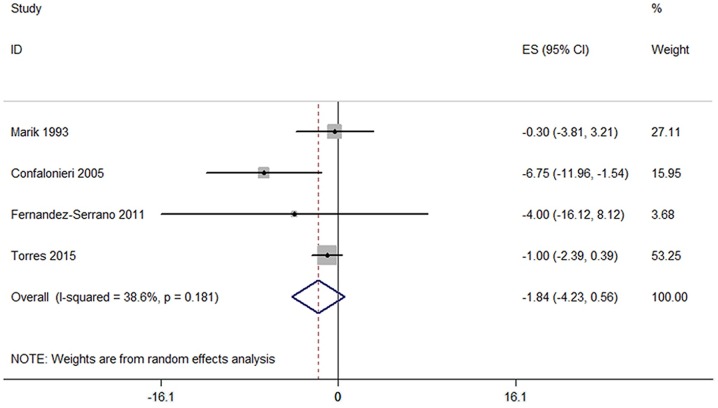
Forest plots of the association between length of ICU stay and corticosteroids.

Based on the data of 4 RCTs, we found that corticosteroids did not significantly decrease the length of ICU stay (−1.84 days, 95% CI:-4.23 to 0.56, p = 0.13, I^2^ = 38.6%) (Fig 5).

#### Acute Respiratory Distress Syndrome

**Fig 6:**
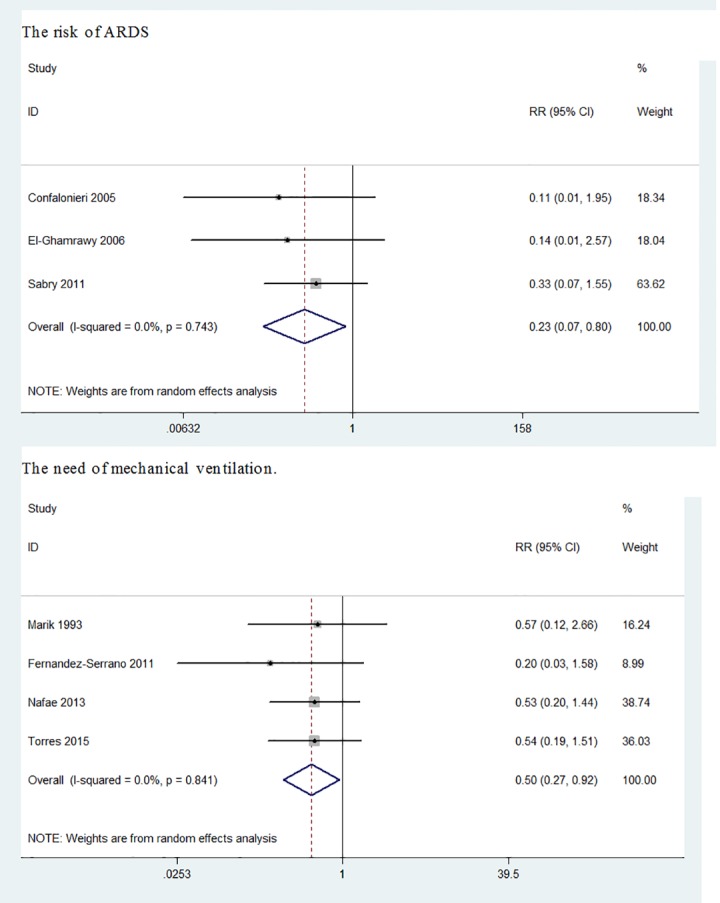
Effect of corticosteroids on development of ARDS and need for mechanical ventilation in patients hospitalized with severe CAP.

3 RCTs evaluated the risk of ARDS in patients. We found a statistically significant reduction in the risk of ARDS treated with corticosteroids (RR = 0.23, 95%CI: 0.07 to 0.80, p = 0.02; I^2^ = 0%) (Fig 6).

#### Mechanical ventilation

4 RCTs revealed a reduction in the need for mechanical ventilation in patients treated with corticosteroids (RR = 0.50, 95%CI: 0.27 to 0.92, p = 0.026; I^2^ = 0%) (Fig 6).

#### Adverse effects

**Fig 7:**
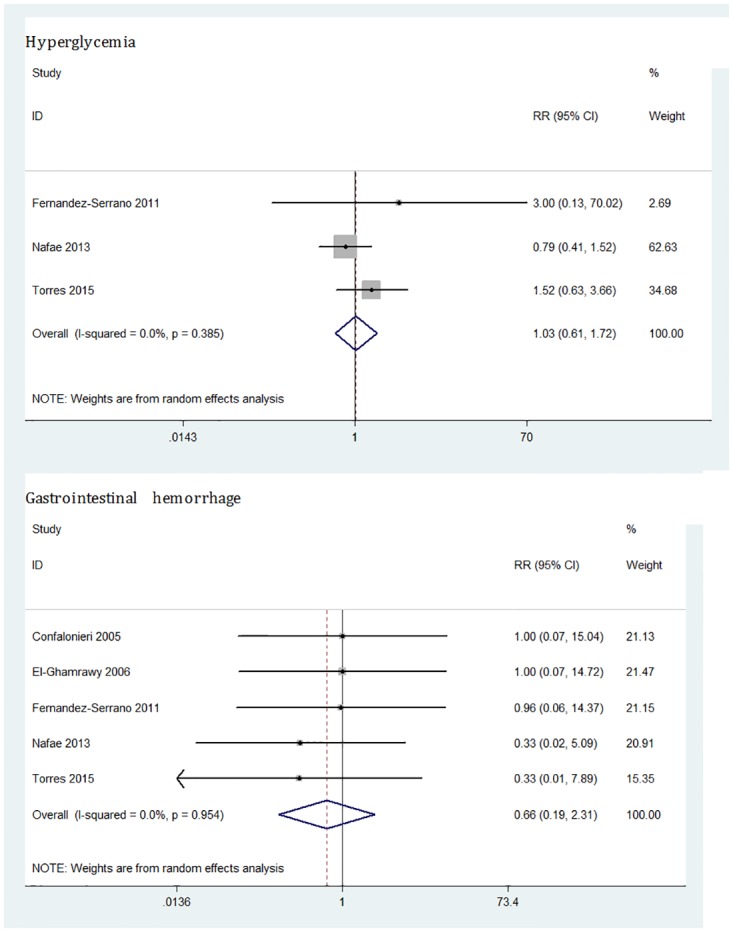
Effect of corticosteroids on hyperglycemia and gastrointestinal hemorrhage in severe CAP patients.

The adverse effects of corticosteroids therapy mainly included hyperglycemia and gastrointestinal hemorrhage. The results of the analysis showed that systemic corticosteroids had no effects on hyperglycemia (3 trials; RR = 1.03, 95%CI: 0.61 to 1.72, p = 0.91; I^2^ = 0%) and gastrointestinal hemorrhage (5 trials; RR = 0.66, 95%CI: 0.19 to 2.31, p = 0.52; I^2^ = 0%) (Fig 7).

### Sensitivity analysis and publication bias

A sensitivity analysis was implemented by the sequential dropping of each study. In above analyses, we just used this analytical method in length of hospital stay.

Considering the limited number (<10) of studies included, we did not evaluate publication bias in this systematic review.

## Discussion

This systematic review and meta-analysis had included 8 RCTs, which evaluated the outcomes of adjunctive corticosteroids therapy in adult patients with severe CAP. The main results of meta-analysis were as follows: (1) corticosteroids reduced all-cause mortality for severe CAP; (2) prolonged corticosteroids therapy shortened length of hospital stay for severe CAP; (3) corticosteroids reduced the risk of ARDS and the need for mechanical ventilation. Additionally, our analysis demonstrated that the incidence of hyperglycemia was increased by systemic corticosteroids but the difference was not statistically significant.

In this meta-analysis, the results demonstrated a statistically significant decrease in the mortality of severe CAP patients treated with corticosteroids. We included all eligible trials with high quality and a low risk of bias except one study [26]. Usually, clinicians used adjunctive corticosteroids therapy for severe CAP especially in the early stage. And there are three potential reasons for the survival advantage. First, corticosteroids are the most powerful inhibitor of inflammation which is part of the complex biological reaction to harmful stimuli such as trauma and pathogens. In addition, the pathogenesis of the majority of severe CAP is usually closely related with the excessive inflammatory response [50]. The anti-inflammatory mechanism of corticosteroids is not completely clear; however, it is believed that corticosteroids play an important role in switching on genes that encode anti-inflammatory cytokines and switching off genes that encode pro-inflammatory cytokines [10, 51]. And in a model of *Pseudomonas aeruginosa* with mechanical ventilation, the results showed that the treatment of antibiotics plus corticosteroids diminished the inflammatory response, decreased bacterial burden in lungs and improved the histopathological severity of pneumonia [52]. Second, the CIRCI was closely associated with severe CAP apart from the excessive inflammation. A systematic review revealed that severe CAP had a prevalence of CIRCI ranging from 0% to 48% [30], and the elevated cortisol levels could be a useful prognostic marker in patients with severe CAP [12]. Therefore, corticosteroids replacement therapy might be effective in severe CAP. Finally, corticosteroids could reduce the mortality of severe CAP for the reason of the reduction in the risk of ARDS and the need for mechanical ventilation [53].

The optimal corticosteroids regimens for severe CAP are not fully explored. The duration of corticosteroids treatment in those studies, which ranged from 1 to 9 days, was five days on average with a median of seven days. Horita et al. found that prolonged use of corticosteroids (>5 days) was not more effective than a short course (≤5 days). To the contrary, our results suggested that prolonged use of corticosteroids (>5 days) was more effective for severe CAP. It is noteworthy that the number of studies refered to the short course of corticosteroids was small (2 studies). Addtionally, The optimal dosage of corticosteroids is also unclear. Most studies used hydrocortisone 200–300mg/day, prednisolone/methylprednisolone 20–50mg/day, or methylprednisolone 1mg/kg/day [35], which were so-called low-or middle-dose of corticosteroids and would be the most suitable dosage in clinical application. Therefore, physicians needed to adjust the dosage and duration of corticosteroids individually.

Our study demonstrated that prolonged use of corticosteroids (>5 days) decreased the length of hospital stay in severe CAP patients. At first, we estimated the result using the value of medians in 5 studies and found a strong heterogeneity (I^2^ = 90.6%). There were three main sources of heterogeneity: methodological heterogeneity, clinical heterogeneity and statistic heterogeneity [47]. In our study, heterogeneity mainly stemed from methodological heterogeneity and clinical heterogeneity, so we conducted a subgroup analysis and sensitivity analysis. Subgroup analysis performed by duration of corticosteroids, the result was -5.79 days (95%CI: -8.17 to -2.31, I^2^ = 61.4%). Considering the study of Nafae with a high risk of bias, we performed a sensitivity analysis to exclude Nafae, and the final result was -4.76 days (95%CI: -8.13 to -1.40, I^2^ = 43.4, p for heterogeneity = 0.17). Adjunctive corticosteroids therapy not only reduced the hospitalization time of severe CAP, but also alleviated the suffering of patients, and finally reduced the financial social-economic burdens [54]. In our study, the potential adverse effects of corticosteroids in severe CAP should be clarified. Theoretically, corticosteroids therapy has the following adverse effects: metabolic disorders (mainly including hyperglycemia), muscle weakness, gastroduodenal bleeding and superinfection. A previous study showed that corticosteroids therapy increased the risks of hypernatremia and hyperglycemia [55]. In our meta-analysis, treatment with corticosteroids in severe CAP was associated with an increased risk of hyperglycemia but the difference had not statistically significant. However, we have not analyzed the association of corticosteroids and the risk of hypernatremia or superinfection, since sufficient information could not be extracted from primary publications. We considered rigorous and comprehensive studies should be designed.

Limitations of this study should be considered: (1) A small number of included studies and relatively small samples had significant influences on the reliability of the results. (2) The definition of severe CAP was not consistent in these studies. It is difficult to achieve a uniform diagnostic criteria due to individual differences in patient, the complex of disease, doctors’ subjective judgments and various severe CAP scoring systems. Therefore, diagnosis of severe CAP in clinical practieces should be comprehensive, included demographic, clinical features plus radiographic, laboratory (some useful biochemical indicators), and microbiologic testing. (3) The opitmal corticosteroids regimens for severe CAP were not fully clarified, which were associated with the differences in type, duration and dosage of corticoteroids, antibiotic therapy, and drug tapering course. (4) The detailed descriptions of severe CAP patients, such as a high inflammatory response, complications, and gender differences, were not fully clear.

In conclusions, our results suggested that adjunctive corticosteroids therapy decreased all-cause mortality in patients with severe CAP, and prolonged corticosteroids treatment was more beneficial for severe CAP. In addition, the study indicated that corticosteroids treatment was safe and reduced the risk of ARDS and the need for mechanical ventilation. Considering the limited number of severe CAP patients, the results may not be stabilized. Thus, large-scale, randomized, double-blind, placebo-controlled trials are needed to evaluate the efficacy and safety of adjunctive corticosteroids therapy in adults with severe CAP.

## Supporting Information

S1 File PRISMA Check List. (PDF)

S2 File Meta-analysis on genetic association studies checklist. (DOCX)

S3 File Detailed search strategy. (DOCX)
