# Supplementary material for: Efficacy and Safety of Adjunctive Corticosteroids Therapy for Severe Community-Acquired Pneumonia in Adults: An Updated Systematic Review and Meta-Analysis
Source: PLoS One. 2016 Nov 15;11(11):e0165942. doi: 10.1371/journal.pone.0165942 (PMC5113003; doi:10.1371/journal.pone.0165942)
Supplement: S3 File — (DOCX) [file pone.0165942.s003.docx]

**Detailed search strategy (Pubmed)**

(("severe"[MeSH Terms] OR "severe"[All Fields]) AND ("residence characteristics"[MeSH Terms] OR ("residence"[All Fields] AND "characteristics"[All Fields]) OR "residence characteristics"[All Fields] OR "community"[All Fields]) AND acquired[All Fields] AND ("pneumonia"[MeSH Terms] OR "pneumonia"[All Fields])) AND (("adrenal cortex hormones"[Pharmacological Action] OR "adrenal cortex hormones"[MeSH Terms] OR ("adrenal"[All Fields] AND "cortex"[All Fields] AND "hormones"[All Fields]) OR "adrenal cortex hormones"[All Fields] OR "corticosteroids"[All Fields]) OR corticotherapy[All Fields] OR ("steroids"[MeSH Terms] OR "steroids"[All Fields]) OR ("dexamethasone"[MeSH Terms] OR "dexamethasone"[All Fields]) OR ("methylprednisolone"[MeSH Terms] OR "methylprednisolone"[All Fields]) OR ("prednisone"[MeSH Terms] OR "prednisone"[All Fields]) OR ("cortisone"[MeSH Terms] OR "cortisone"[All Fields]) OR ("hydrocortisone"[MeSH Terms] OR "hydrocortisone"[All Fields]) OR ("prednisolone"[MeSH Terms] OR "prednisolone"[All Fields]))
